# Efficacy of Disease Modifying Therapies in Progressive MS and How Immune Senescence May Explain Their Failure

**DOI:** 10.3389/fneur.2022.854390

**Published:** 2022-03-31

**Authors:** Navid Manouchehri, Victor H. Salinas, Negar Rabi Yeganeh, David Pitt, Rehana Z. Hussain, Olaf Stuve

**Affiliations:** ^1^Department of Neurology, The University of Texas Southwestern Medical Center, Dallas, TX, United States; ^2^Department of Radiopharmacy, Faculty of Pharmacy, Tehran University of Medical Sciences, Tehran, Iran; ^3^Department of Neurology, Yale University, New Haven, CT, United States; ^4^Neurology Section, VA North Texas Health Care System, Medical Service Dallas, Veterans Affairs Medical Center, Dallas, TX, United States

**Keywords:** adaptive immunity, innate immunity, multiple sclerosis, immunosenescence, progressive multiple sclerosis, disease modifying therapies

## Abstract

The advent of disease modifying therapies (DMT) in the past two decades has been the cornerstone of successful clinical management of multiple sclerosis (MS). Despite the great strides made in reducing the relapse frequency and occurrence of new signal changes on neuroimaging in patients with relapsing remitting MS (RRMS) by approved DMT, it has been challenging to demonstrate their effectiveness in non-active secondary progressive MS (SPMS) and primary progressive MS (PPMS) disease phenotypes. The dichotomy of DMT effectiveness between RRMS and progressive MS informs on distinct pathogeneses of the different MS phenotypes. Conversely, factors that render patients with progressive MS resistant to therapy are not understood. Thus far, age has emerged as the main correlate of the transition from RRMS to SPMS. Whether it is aging and age-related factors or the underlying immune senescence that qualitatively alter immune responses as the disease transitions to SPMS, that diminish DMT effectiveness, or both, is currently not known. Here, we will discuss the role of immune senescence on different arms of the immune system, and how it may explain relative DMT resistance.

## Introduction

Multiple sclerosis (MS) is the most prevalent inflammatory disorder of the central nervous system (CNS) with a presumed autoimmune pathogenesis. MS was traditionally viewed as a T cell-mediated inflammatory disorder based on numerous observations made over the span of many decades. Aside from the abundance of lymphocytic infiltrates in MS lesion biopsies, other factors included: (A) the induction of the experimental autoimmune encephalomyelitis (EAE) model of MS in healthy recipient animals by adoptive transfer of myelin-reactive CD4^+^ T helper (Th) cells from previously immunized donor mice (1); (B) the genetic association of MS with human leukocyte antigen (HLA) DRB1^*^15:01 (2), a major histocompatibility complex (MHC) class II molecule, required for the presentation of linearized peptides to CD4^+^ Th cells; (C) the failed attempt to treat MS patients with an altered peptide ligand of myelin basic protein (MBP) that activated MBP-reactive CD4^+^ Th cells, leading to disease exacerbation instead (3); (D) the initiation and re-activation of MS with immune checkpoint inhibitors during cancer therapy (4); and (E) the beneficial effects of pharmacotherapies in early relapsing MS that deplete T cells, or sequester them out of the CNS (5, 6). This last aspect has illustrated how in early MS, relapses and new MS brain lesions are triggered and perpetuated by T cells and possibly other bone marrow-derived immune cells (7). The success of B cell depleting therapies in treating active MS, further corroborates the role of bone marrow-derived immune cells outside of the T cell compartment in pathogenesis of the disease (8–10). Changes in the clinical phenotype of MS, including treatment responsiveness will likely be linked to these cells as well.

A clinical course typified by relapses followed by periods of remission defines relapsing-remitting MS (RRMS) (11, 12). Patients with early MS who display clinical and paraclinical magnetic resonance imaging (MRI) disease activity gain a detectable and substantial benefit from receiving disease modifying therapies (DMT); patients without these evidences of disease activity, are defined as progressive MS (PMS); specifically, based on the 2013 Lublin criteria, PMS patients accrue objectively documented neurological disability without intermittent recovery and do not appear to receive any benefit from DMT (12, 13). Thus, the molecular and cellular signature of MS, as the primary therapeutic targets, change with age and disease becomes non-active. PMS at this stage is considered either secondary MS (SPMS) when following a period of RRMS, or primary progressive MS (PPMS) in lieu of relapsing disease activity (11, 12). PPMS patients are ~10 years older upon diagnosis than RRMS patients. A subsection of patients with PMS, do show disease activity as defined above (12). There is no disease biomarker to indicate when the transition from RRMS to SPMS starts or is completed.

Currently, different hypotheses try to explain this transition; to date, age has been the most relevant prognostic factor underlying the transition from active RRMS to non-active SPMS (14–17). In contrast, a meta-analysis of all blinded, randomized clinical trials of DMT for RRMS indicated that DMT efficacy were independent of the recipients' age (18) despite a clear trend toward reduced effectiveness. Unfortunately, individualized data was not made available to the authors, and these results have to be interpreted with some caution.

As a biological correlate to age, immunosenescence has been advocated as a candidate to explain diminished DMT efficacy in PMS (19). Immunosenescence correlates with age relative to overall life expectancy (20). It is often accompanied by a decline in key immune functions such as the capacity for strictly non-self-antigen presentation and breadth of antigen recognition, the formation of long-lasting immune memory, and active immune surveillance (20). Here, we discuss whether immunosenescence contributes to the transition from active to non-active MS and how that correlates to loss of DMT efficacy in the context of PMS.

## Age, Immunosenescence, and Loss of DMT Efficacy

The transition to SPMS may not be entirely influenced by age. It takes place on average two decades following the clinical diagnosis of RRMS in adult-onset MS (AOMS) (21). Given that currently approved DMT remain effective even in patients with late-onset MS (LOMS), diagnosed in 50-year-old patients or older, it appears counterintuitive to attribute DMT unresponsiveness to age alone. Although, PPMS is more prevalent among LOMS than AOMS, still, nearly 50% of LOMS cases are RRMS and respond to DMT (18, 22). Immunosenescence as a potential contributor to DMT-resistance may be present in both AOMS and LOMS, and not entirely driven by age.

A correlate of intact adaptive immune function is responsiveness to vaccination with neo-antigens. Expectedly, vaccine efficacy wanes in elder populations (23); however, data on vaccine response among elderly RRMS demonstrate substantial adaptive immune response, despite long term DMT treatment with proven negative effects on vaccine response (24), reiterating how age alone does not define quality of immune response.

Current DMT in MS, including interferons (25–27), copolymers (28) depleting agents against CD20 (8–10) and CD52 (29), nucleoside synthesis antagonists (30–32), sphingosine-1 phosphate receptor modulators (33–36), nuclear 1 factor (erythroid-derived 2)–like 2 modulators (37) or α4-integrin antagonists (38, 39), aim to either deplete lymphocytes, modulate pro-inflammatory features or inhibit their traffic into the CNS. These DMT classes are approved for use in active MS. The validating trials for the only two FDA-approved DMT for use in SPMS, namely, cladribine (40, 41) and siponimod (34) recruited a mix of active and non-active progressive MS patients, limiting their relevance in pure non-active MS cohorts (13). These trials likely benefited participants with residual active MS (42). Conceivably, immunosenescence, both predates and promotes the transition from DMT-responsive active MS to DMT-resistant non-active MS. However, instigators or accelerators of immunosenescence in the context of MS require further elucidation.

## Adaptive Immunosenescence and DMT-Resistant PMS

**Figure 1:**
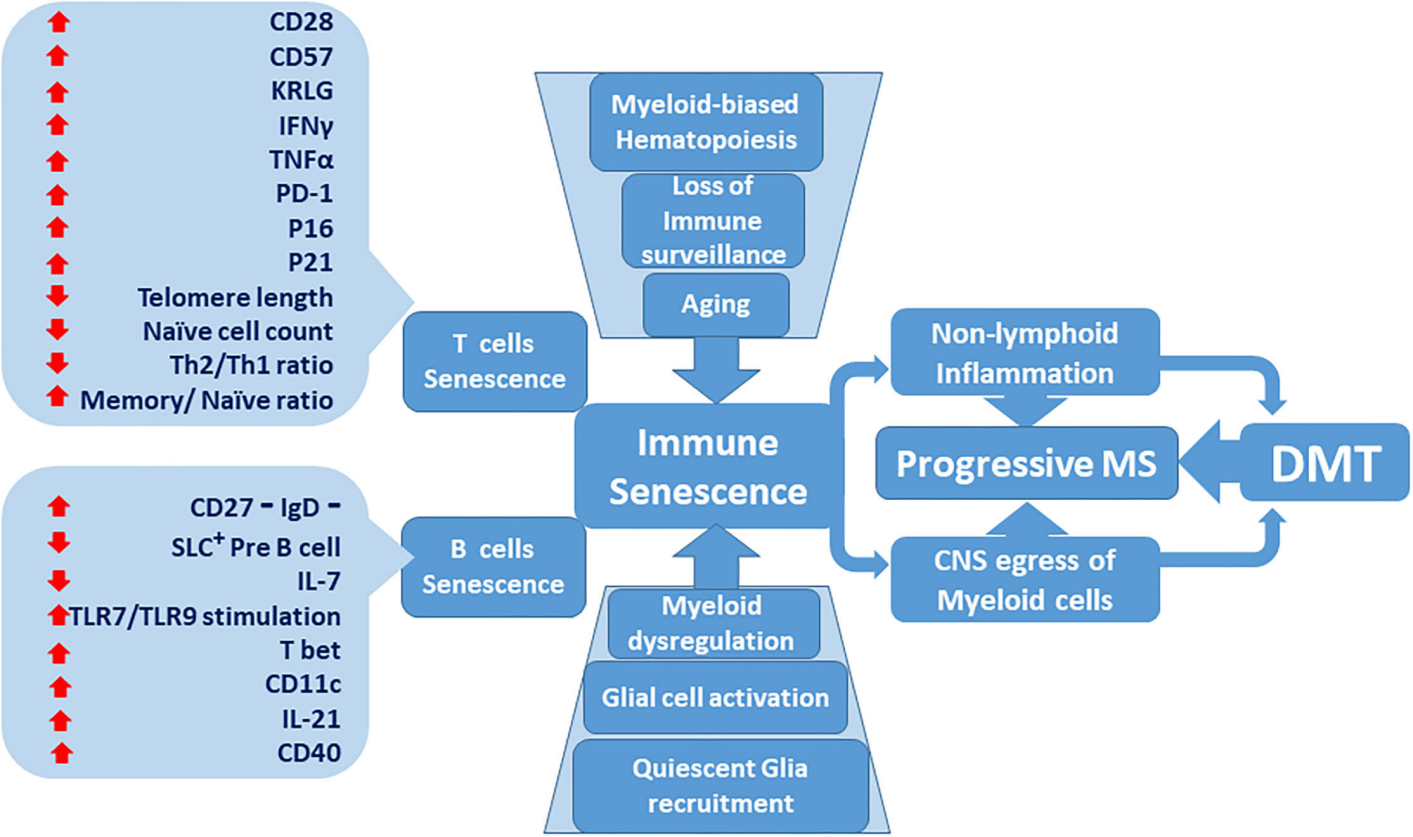
Immune senescence contribution to the lack of effectiveness of disease-modifying therapies (DMT) in progressive multiple sclerosis (MS). Senescent T and B immune cell phenotypes become more predominant as the immune system ages. Age-dependent deterioration of tissues including central nervous system (CNS) glial cells increases. Bone marrow-derived myeloid cells are activated and compound adaptive immune senescence. Dysregulated myeloid cells prospectively accumulate within the CNS milieu. Inflammatory MS, characterized by active relapses, transitions to non-active progressive MS, which is resistant to DMT.

The adaptive immune system is not fully competent at birth; it becomes fully functional post-puberty and in early adulthood, declining progressively thereafter (43–46). Despite the age-associated decline in thymic epithelial tissue, it has been demonstrated that both the thymic cortex and thymic medulla function throughout life (47–49); however, the inevitable thymic involution is accompanied by the reduction of T cell diversity (49). Intact and functional thymic epithelium continually produces T cells migrating out of thymic medulla to peripheral lymphoid organs (50, 51). T cells generated from thymopoiesis have a full T cell receptor (TCR) repertoire, and are capable of generating responses to neo-antigens. In contrast, expansion of the peripheral T cells, driven by thymic involution may lead to repertoires limited to those of existing memory T cells and reduced capacity of immune response to new antigens (52–55). As mentioned, T cells are critical in initiating and perpetuating inflammation in active MS (7). MS pathogenesis potentiates T cell-antigenic spreading and repeatedly stimulates CNS-specific T cells (56). Suppressed CD28 expression, mediated by repeated antigenic stimulation is associated with senescent phenotypes in T cells (Figure 1) (23, 57–61). A similar phenotype of CD28^low^ T cells is detectable in the pool of circulatory effector memory T cells with senescent attributes in the context of MS. (62, 63). Immunosenescence is not restricted to cellular immunity and similarly carries over to B cell-mediated immune responses (61). Noticeably, non-cellular adaptive immune responses mediated by B cells have been implicated by preclinical models as mediators of CNS autoimmunity (64). The Epstein-Barr virus (EBV), a plausible pathogenic MS trigger, is in fact a B cell tropic infection (65). EBV infection primes polyclonal populations of B cells to avidly present self-antigens to autoreactive CD8^+^ cytotoxic T cells (66). Post-mortem studies that have yet to be reproduced showed abundant EBV infected B cells within actively demyelinating MS lesions (66, 67). Similar to T cells, constant B cell activation may drive premature senescence (68). Interestingly, surface expression of CD40, a correlate of B cells antigen presentation and memory formation, signifies senescence and is elevated in EBV infected B cells (69). In B cell senescence, it is quality rather than quantity of humoral response that declines, resulting in comparable volume of antibodies, albeit less effective. This is evident from diminished antibody specificity for foreign antigens, and decreased predominance of IgG isotypes along with lowered affinity of antibodies (70). Prematurely senescent B cells provide antigen presentation for expansion and maintenance of T cells in autoimmune disease like MS. Namely, increased signaling via CD80, CD86, CD11c, and CD40 by B cells in MS patients is higher than healthy controls and responsible for promotion of inflammatory T cell responses. Ultimately, the expression of these markers correlates with exhausted or senescent B cell phenotypes (Figure 1) (23, 57–61, 71). As discussed earlier, anti-CD20 agents that deplete B cells have become a mainstay of therapy for active MS with proven efficacy (72). However, B cell depletion with the humanized anti-CD20 monoclonal antibody (72) ocrelizumab, which is approved for PPMS, does not deliver significant neuroprotective effects as assessed by serial blood measurements of neurofilament light chain (NfL) (73). CD20^+^ cells are not a single population and range from naïve B cells to fully matured memory cells. Since production of new pro-B cells is directly affected by senescence (74), continued treatment with anti-CD20 therapies likely depletes naïve B cells with diverse B cell receptor (BCR) repertoires. Likewise, continuation of T cell-depleting DMT in non-active PMS will likely result in T cells that lack diverse naïve cells. In this manner, increasing age and DMT therapy compound adaptive immunosenescence in MS. Nevertheless, if adaptive immunity was relevant in perpetuating disease progression in PMS, DMT effectiveness should have remained constant or even slightly improved since PMS is dominated by senescence. In fact, if DMT unresponsiveness in PMS was driven predominantly by adaptive immunity, decreased potency of senescent adaptive immunity would have led to improvement of clinical outcomes in elder patients. Since DMT optimally address adaptive immune cells in the periphery, DMT resistance in non-active PMS requires explanations beyond adaptive immune components or peripheral compartment.

## Innate Immunosenescence and DMT Resistance in PMS

Innate immunity is influenced by the immunosenescence. Contrary to adaptive immunity, the innate immune system in mammals is considered functional at birth, and retains most of its function throughout life (61). Cell migration, adhesion and phagocytosis of polymorphonuclear leukocytes (PMN) were believed to stay virtually unaltered by aging (75–78); however studies have shown that certain innate immune cell functions may falter with age (79–81). Immunosenescence pushes innate immune cells toward functional dysregulation that compounds the effects of suboptimal senescent adaptive immunity. In MS, effects of immunosenescence on innate immune are noticeable in four domains; namely, (1) Aged innate immune system manifests with the preponderance of dys-homeostatic phenotypes and *forme pleine* of the ideally self-limiting responses. This is likely to promote chronic and continued tissue destruction, sub-functional remodeling and delayed healing (81–84). (2) Within the CNS, myeloid cells, as antigen presenting cells (APC), re-activate and retain CD4^+^ T cells, and contribute to effective immune surveillance (85). These cells are altered via senescence. There are three compartments within the brain where myeloid cells exert their effect: (A) the parenchyma, (B) cerebral perivascular spaces (CPVS) and (C) meninges. Parenchymal microglia are tissue-intrinsic macrophages of the CNS (86–88). The other relevant compartments for antigen presentation are CPVS and meninges, which are populated by monocyte-derived macrophages and dendritic cells (DC). In CX_3_CR_1_ GFP^+^ mice, it was demonstrated that microglia and monocyte-derived brain macrophages are distinct entities (89, 90). Meninges have been implicated as an anatomic site in host defense and autoimmunity. Major histocompatibility complex II (MHCII)-positive cells, detectable within all meningeal layers (91), include monocyte-derived macrophages, monocyte-derived DC (mDC) and classical DC (cDC) (92). While some studies reported DC from young and aged humans having similar surface expression of MHCII molecules and elicited equal T cell proliferation, other investigators demonstrated significantly lower MHCII expression by DC in the elderly (93, 94). (3) During hematopoiesis, immunosenescence reduces lymphopoiesis in favor of heightened myelopoiesis, resulting in a net increase in myeloid cell output (59). (4) Inflammaging, defined as chronic, low grade and sterile inflammation, despite the overall diminishment in immune functions, increases with age (81, 95). Bone marrow-derived myeloid cells (BMC), activate and upregulate surface adhesion molecules in response to inflammation (96). This may allow BMC to penetrate and accrue within target tissues such as CNS (97).

CNS microenvironment in response to the aforementioned events may shift to a dys-homeostatic state adopted by CNS-resident myeloid cells (97). Previous observations postulated that compared to naïve quiescent microglia, activated microglia and infiltrated BMC during CNS inflammation upregulate inflammation-associated signals (92). Myeloid cells within the CPVS are constantly replaced by BMC, and this turnover is accelerated during inflammation (98). During EAE, parenchymal microglia and BMC exhibited mutual activation markers following the onset of clinical disease in mice. Specifically, clinical disease onset was temporally associated with the appearance of BMC in the CNS inflammatory milieu (97). This was not a transient event and the newly present activated BMC were likely retained within the CNS microenvironment and merged with the activated microglial pool (97). It is still unclear to what extent these changes advance functional disarray and homeostatic disturbance; however, current DMT in MS do not primarily target BMC. Certain DMT might modulate the trafficking of BMC into the CNS, as demonstrated with natalizumab therapy (99). However, the ASCEND trial, a phase 3 study on the efficacy of natalizumab therapy in SPMS, failed to show meaningful clinical benefits (100), perhaps suggesting that ongoing migration of BMC in SPMS is no longer highly relevant to disease progression.

“Smoldering” MS lesions are a candidate to explain PMS and chronic destruction of CNS parenchyma without evident inflammation. They are dominated by the presence of perilesional activated myeloid cells without lymphoid inflammation (101, 102). Whether these myeloid cells are CNS intrinsic or bone marrow-derived, or a mixture of both is incompletely understood. Smoldering MS is more prevalent among SPMS patients who are on average older than active MS patients, however, as we discussed before, age likely is not the driving factor. The transition to SPMS takes place faster in LOMS patients; however, accrual of disability for LOMS patients during years living with SPMS is slower in comparison (21). Possibly, later onset and relatively brief inflammatory phase in LOMS, spares them from fully-recognized disease burden. In the context of non-active PMS, antigen-independent and compartmentalized chronic inflammation, shielded from therapeutic efforts, is plausible. The corollary to this hypothesis would be that even outside of common DMT, other anti-proliferative therapies might fail to provide meaningful clinical benefit to PMS (103), a logic that should guide hematopoietic stem cell transplants as well (104). Furthermore, if myeloid cells drive PMS, they probably do so in a stage-specific fashion; (1) BMC likely enter the CNS throughout active and non-active MS and are retained within the CNS; and (2) altered CNS-intrinsic myeloid cells, respond to BMC presence, at sites similar to the border of smoldering lesions. Adaptive immune cells most likely instigate these events at first; during non-active MS, such external cues may no longer be absolutely required and suppression of adaptive immune system *via* DMT, thereafter provide little clinical benefit.

## Glial Senescence and DMT Resistance in PMS

Glial cells including astrocytes and microglia are critical in CNS. Immune functions related to astrocytes in the context of MS and its preclinical models have recently attracted renewed interest; however their pathogenic role in MS is currently less clear. For instance, the depletion of astrocytes worsens clinical disease in an acute EAE model but ameliorates progressive EAE (105). A subpopulation of astrocytes expresses complement component 3 (C3) in response to interleukin-1 alpha (IL-1α) tumor necrosis factor alpha (TNFα) and complement component 1, subcomponent q (C1q), and possesses neurotoxic properties and are upregulated in MS lesions (106). Their development underscores a bi-directional interaction between astrocytes and myeloid cells. Pro-inflammatory signals from Microglia and BMC in CNS are a primary inducer of such astrocytes while activated astrocytes allow further recruitment and entry of pro-inflammatory monocytes to the CNS; this multiplicative effect may have been intercepted by astrocyte depletion leading to the aforementioned amelioration of chronic EAE (106, 107). Furthermore, senescent astrocytes, may contribute to neurodegeneration in an overburdened neural network post demyelination. Astrocytes possess star-shaped appearance, and are intimately associated with the CNS vasculature (108). Together with endothelial cells and pericytes, astrocytes are critical in forming and maintaining the blood brain barrier (BBB). Astrocytes can express MHC class II molecules in defined experimental conditions which endows them to serve as potential APC to CD4^+^ T helper cells (109–111). Cellular changes associated with astrocyte senescence include the increased expression of glial fibrillary acidic protein (GFAP) and vimentin (112, 113). This is at least partly driven by increased signaling of transforming growth factor beta 1 (TGFβ1). TGFβ1 inhibits astrocyte proliferation and induces a senescence-associated secretory phenotype (SASP), which involves an enhanced expression of inflammatory molecules (19, 114).

Inflammation induced phenotypes in microglia also mimic senescence. Specifically, CNS microglia exhibit a phenotypical profile that has been frequently associated with aging in the context of neurodegenerative disorders (115). These attributes, including increased iron storage, production of pro-inflammatory cytokines, lower motility and diminished phagocytic capacity are not strictly age-dependent and are inducible by other insults as well. Single cell transcriptomic studies on EAE models as well as human samples have shown presence of distinct microglia-like cells in CNS inflammation (97, 116). Microglia are maintained by local proliferative self-renewal (90). Within the CNS, functional properties of microglia, as tissue resident myeloid cells, across age groups are likely constant. Interestingly, it was recently shown that microglial density in the brain increases in aged mice (117). However, the authors had utilized markers that correlated with activated microglia, namely Iba-1. Therefore, the observed increase in microglia density with age might point more toward heightened microglial activation and subsequent reduction in the pool of homeostatic quiescent microglia. Senescent microglia are found within the brains of PMS patients despite pronounced reduction in inactive lesions (118, 119). As discussed, these cells might exert their role at the border of smoldering lesions (102). Phagocytosis of myelin debris supports re-myelination efforts; microglial depletion associates with loss of phagocytic capacity in the microenvironment of MS lesions, likely promoting dysmyelination. Aged human microglia exhibit proclivity to express ferritin, believed to be associated with senescence (120). Increased iron uptake likely follows the destruction of iron-containing oligodendrocytes in MS and is observable in aged microglia. These limitations to CNS re-myelination efforts, possibly lend to PMS phenotype.

Glial cells are not a primary target for current DMT, and their role in relation to why DMT fail remains to be explained. The restricted CNS bioavailability of most DMT, is unlikely to impact glial cells. Further studies are required to elucidate the significance of these observations and their potential for development of novel therapeutics.

## Toward a Theory for DMT Resistance in Progressive MS

Based on extensive data from clinical trials and post-approval observational studies it is evident that the current therapeutic dogma in MS, namely the depletion of inflammatory adaptive immune cells or their sequestration out of the CNS is effective during active RRMS. Current data gleaned from clinical trials suggest that most DMT have minimal effects on non-active progressive MS without signs of disease activity (13, 34, 73, 121, 122); therefore administration of DMT that target immune components may not be in the best interest of these patients. Alternatively future therapeutic endeavors may benefit from incorporating strategies that cover innate immunity and glial targets within the CNS.

The evidence presented here supports a view of DMT resistance associated with immunosenescence in PMS (Figure 1). Possible pharmacological efforts to address immunosenescence may adopt designs that identify senescence-specific factors, amenable to modulation. A desired goal and measure of success could be delayed transition to non-active PMS. Current DMT, as effective as they are in controlling RRMS, are unqualified to address the breadth of ongoing deleterious effects of MS (123–125), including the role of innate immune cells and CNS glial components. Myeloid cells, as potential candidates for targeted novel therapies in non-active PMS, retain phenotypical plasticity. Curtailing the dys-homeosatic signaling in these cells and safeguarding against further disruption of CNS quiescence, is a biological plausibility (126, 127). Given the current availability of analytic tools with single-cell resolution, deep characterization of myeloid sub populations and definition of exclusive phenotypical signatures in pertinent compartments is both feasible and indispensable. Molecular targets, acquired through this process could serve as a map for pre-clinical efforts (128, 129).

In conclusion, immunosenescence as a multivariate phenomenon, is not defined by advancing age alone and its constellation of immunological effects over time culminates to DMT resistance in PMS. Current DMT mechanisms of action are optimized to mitigate inflammation-induced damage during active MS. They are limited to address the inevitable transition to non-active PMS or remain effective thereafter. Clinical and biological data call for a more targeted approach with an emphasis on myeloid cells, innate immunity components and glial cells in the future.

## Author Contributions

NM and NR surveyed the literature. NM, VS, and NR drafted the manuscript. RH, DP, and OS provided expert opinion and edited the final manuscript. All authors contributed to the article and approved the submitted version.

## Funding

OS was a 2021 recipient of a Grant for Multiple Sclerosis Innovation (GMSI), Merck KGaA, and was funded by a Merit Review grant (federal award document number (FAIN) BX005664-01 from the United States (U.S.) Department of Veterans Affairs, Biomedical Laboratory Research and Development). The funders were not involved in the study design, collection, analysis, interpretation of data, the writing of this article or the decision to submit it for publication.

## Conflict of Interest

OS serves on the editorial boards of Therapeutic Advances in Neurological Disorders, has served on data monitoring committees for Genentech-Roche, Pfizer, Novartis, and TG Therapeutics without monetary compensation, has advised EMD Serono, Celgene, Genentech, Genzyme, TG Therapeutics, and VYNE, receives grant support from EMD Serono and Exalys, was a 2021 recipient of a Grant for Multiple Sclerosis Innovation (GMSI), Merck KGaA, and was funded by a Merit Review grant (federal award document number (FAIN) BX005664-01 from the United States (U.S.) Department of Veterans Affairs, Biomedical Laboratory Research and Development). The remaining authors declare that the research was conducted in the absence of any commercial or financial relationships that could be construed as a potential conflict of interest.

## Publisher's Note

All claims expressed in this article are solely those of the authors and do not necessarily represent those of their affiliated organizations, or those of the publisher, the editors and the reviewers. Any product that may be evaluated in this article, or claim that may be made by its manufacturer, is not guaranteed or endorsed by the publisher.
